# Piezoelectric energy extraction from a cylinder undergoing vortex-induced vibration using internal resonance

**DOI:** 10.1038/s41598-023-33760-5

**Published:** 2023-04-28

**Authors:** Annette Joy, Vaibhav Joshi, Kumar Narendran, Ritwik Ghoshal

**Affiliations:** 1 Department of Ocean Engineering and Naval Architecture, Indian Institute of Technology Kharagpur, Kharagpur, 721302 India; 2 Department of Mechanical Engineering, Birla Institute of Technology and Science Pilani, K K Birla Goa Campus, Sancoale, Goa 403726 India; 3 Department of Ocean Engineering, Indian Institute of Technology Madras, 600036 Chennai, India

**Keywords:** Devices for energy harvesting, Hydroelectricity, Wind energy, Mechanical engineering, Fluid dynamics

## Abstract

A novel concept of utilizing the kinetic energy from ocean currents/wind by means of internal resonance is proposed to address the increasing global energy demand by generating clean and sustainable power. In this work, a non-linear rotative gravity pendulum is employed to autoparametrically excite the elastically mounted cylinder for a wide range of flow velocities. This concept is adopted to increase the oscillation amplitude of the cylinder due to vortex-induced vibration (VIV) in the de-synchronized region for energy harvesting. In this regard, a VIV-based energy harvesting device is proposed that consists of a cylinder with an attached pendulum, and energy is harvested with bottom-mounted piezoelectric transducers. The cylinder undergoes VIV when it is subjected to fluid flow and this excites the coupled fluid-multibody cylinder-pendulum system autoparametrically. In the de-synchronized region, when the vortex shedding frequency becomes two times the natural frequency of the pendulum, an internal resonance occurs. This helps in achieving a higher oscillation amplitude of the cylinder which does not happen otherwise. This study is focused on the two degree-of-freedom (2-DoF) cylinder-pendulum system where the cylinder is free to exhibit cross-flow vortex-induced vibrations subjected to the fluid. The objective of this work is to numerically investigate the effect of a non-linear rotative gravity pendulum (NRGP) on the VIV characteristics and piezoelectric efficiency of the system. The numerical model is based on the wake-oscillator model coupled with the piezoelectric constitutive equation. The influence of the frequency ratio, mass ratio, torsional damping ratio, and ratio of cylinder diameter to pendulum length of the NRGP device on response characteristics due to VIV is also investigated. A detailed comparative analysis in terms of electric tension and efficiency is performed numerically for flows with a wide range of reduced velocities for the cylinder with and without NRGP. A comprehensive study on the implications of internal resonance between the pendulum and a cylinder undergoing VIV on generated electric tension is also reported.

## Introduction

Vortex-induced vibrations (VIV) are one of the most common hydrodynamic phenomena with practical implications that can be observed when the structures are subjected to fluid flow. VIV has been studied in detail by a number of investigators such as Roshko^1^, Griffin and Ramberg^2^, Bearman^3^; in review articles of Williamson and Govardhan^4^, Sarpkaya^5^ and in books by Belvins^6^, Sumer and Fredsøe^7^. Over the past few decades, many researchers have focused on different methods to harness the hydro-kinetic energy utilizing the vortex-induced motion of structures and convert it into electrical energy^8,9^. The VIV of structural components can be converted to electrical power using electrostatic^10^, electromagnetic^11^ and piezoelectric generators^12^ which can be used to power micro-electro-mechanical systems or for charging batteries in remote locations. These small-scale energy-generating sources are useful in powering nearby electronic equipment and self-powered devices^13^. It should be noted that, in a real VIV problem, the electromechanical systems are subject to the effects of ambient noise, i.e., fluctuations in incoming flow or geometrical imperfections of the system and it can significantly influence the dynamic behaviour. Therefore, for efficient energy harvesting the effects of different stochastic noises are also being investigated by various researchers^14,15^.

In recent years, there are numerous contributions focusing on efficient ways of extracting energy from VIV using piezoelectric transducers. These transducers have a unique capability of converting strain energy to electrical energy. The most common and easiest way to extract energy is by attaching the piezoelectric material to the flexible/elastically mounted structure. Truitt^16^ devised a wind-based energy harvester, by fixing a Polyvinylidene fluoride (PVDF) piezoelectric material on a flag-like membrane, and obtained a maximum power of 1.5 mW. Song et al.^17^ proposed a novel concept of energy harvesting utilizing VIV and wake-induced vibrations (WIV) of two tandem cylinders connected by piezoelectric membranes as cantilevers and recorded a maximum power output of 21 $\mu$W. Wang and Ko^18^ harvested energy from a piezoelectric film fixed over the fluid flow channel. Numerical investigations were conducted by Mehmood et al.^19^ by using electromechanical governing equations that couple the oscillation of an elastically mounted cylinder fixed with piezoelectric material. They observed that there is a significant impact on synchronization width and amplitude due to load resistance. Franzini and Bunzel^20^ carried out numerical investigations on the power output from cylinders mounted on piezoelectric harvesters subjected to VIV. In their study, two different configurations pertaining to uni-directional (cross-flow) and bi-directional (cross-flow and in-line) VIV were studied. In both configurations, power output and efficiency were higher when the frequency of vortex shedding was near the structural frequency, i.e., in the lock-in region. A maximum power output of 2.6 mW and 11 mW for the uni-directional and bi-directional VIV were reported, respectively. Experimental investigations were conducted by Arionfard and Nishi^21^ for a pivoted cylinder undergoing VIV for Reynolds number (*Re*) ranging from 2880 to 22300 and reported a maximum power output of 60 mW. In a subsequent experimental study, Nishi et al.^22^ proposed an efficient way of extracting energy by placing a secondary cylinder between the generator and the primary cylinder exposed to VIV, which increased electric tension (voltage) up to 9 V. In a numerical investigation, Soti et al.^23^ reported that attaching the cylinder to a magnet can give a maximum harvested dimensionless power up to 0.13 at $Re = 150$. The energy harvesting was also investigated on a cross-flow vibrating circular cylinder with a secondary mass spring mounted upon it forming a two-degrees of freedom (2-DoF) system in Lu et al.^24^ Two “lock-in” regions were observed in this system corresponding to the first- and second-order resonances of the system. Theoretical analyses were performed in the works by Hu et al.^25,26^ on a 2-DoF system to assess energy harvesting capabilities of galloping as well as concurrent aeroelastic and base excitation. These studies were carried out from an aeroelastic perspective dealing with high mass ratios. However, flow-induced effects become more challenging to analyse for low mass ratios, typically observed in marine and hydrodynamic environments. A detailed discussion regarding the recent developments of various devices for piezoelectric energy harvesting can be found in review articles by Elahi et al.^27^.

Recently the possibility of energy harvesting from parametrically forced pendulum has gained attention among many researchers^28–30^. Marszal^31^ conducted both experimental and numerical methods to harvest energy from pendulum oscillations using a generator and reported that energy harvesting was more efficient for shorter pendulum lengths. Franzini and his co-workers^32,33^ in a series of numerical investigations highlighted that parametric excitation can significantly influence energy harvesting. However, in most of the studies, the effect of the pendulum on the base structure was neglected. In successive publications, Das and Wahi^30,34,35^ presented the feasibility of extracting energy from vortex-induced vibrations by controlling the rotating motion of an attached pendulum, wherein an attempt was made to get an insight into the system dynamics through the method of multiple scales (MMS), harmonic balance (HB), continuation methods, etc. The coupling effect of the pendulum on the base structure was considered in their work^35^ and it was concluded that the response was significantly influenced by pendulum rotation. Both vertical and horizontal configuration of the pendulum was considered in their study, however, no quantitative difference in terms of electrical power and efficiency were reported. To the best of the authors’ knowledge, the influence of attaching a non-linear rotative gravity pendulum on harvested electrical power and a detailed study on parametric/autoparametric resonance of this type of multibody system is yet to be explored.

Although energy extraction from ocean currents from VIV is not unfamiliar, in this work, the effect of attaching a non-linear rotative gravity pendulum (NRGP) on a VIV-based energy harvesting device is studied. The main contribution of this paper is to illustrate the effect of 2:1 internal resonance on harvested electrical power. In this regard, the dynamics of a rigid cylinder with NRGP mounted on elastic support having piezoelectric harvesters, is studied numerically in a cross-flow VIV set-up. A non-linear wake-oscillator model by Ogink and Metrikine^36^ is used for estimating the fluid load modified from the original methodology of Facchinetti and de Langre^37^. The coupling of the solid-electric multibody system is modelled through a linear constitutive equation. In this article, mathematical models for the NRGP-VIV system coupled with a piezoelectric harvester (PZH) device are presented and numerical simulations are performed to obtain oscillation amplitude, electric tension, and time-averaged electric power. Results are compared with existing numerical models and experiments on similar devices. A detailed sensitivity study on 2:1 internal resonance and its influence on harvested electrical power is also presented.

The rest of the article is organized as follows: In Sect. "Coupled problem description and methodology", governing differential equations for cross-flow VIV based on the wake-oscillator model and the equation of motion of the autoparametric cylinder-pendulum system are discussed along with the problem definition. The numerical results obtained from the formulation are compared with the literature in Sect. "Comparative study". The effect of the introduction of the NRGP on the amplitude response of the cylinder and piezoelectric harvesting capability are also discussed. A detailed parametric analysis of the coupled NRGP-PZH-VIV system is performed in Sect. "Parametric study of the NRGP-PZH-VIV system". Finally, the concluding remarks on the efficacy of the proposed autoparametric oscillator based energy harvesting device are given in Sect. "Conclusions".

## Coupled problem description and methodology

**Figure 1:**
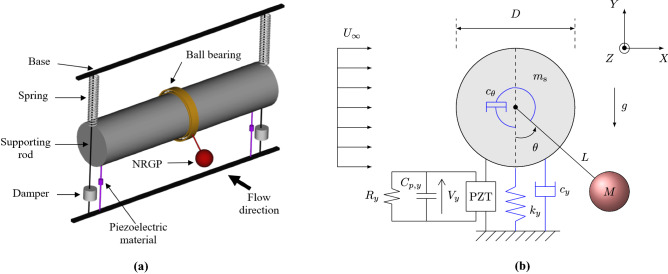
A VIV-based energy extraction device with the NRG pendulum. (**a**) 3-D illustration of the conceptual model, and (**b**) Schematic description.

We begin by briefly describing the governing equations of the non-linear rotative gravity pendulum based vortex-induced vibration (NRGP-VIV) amplifier coupled with the piezoelectric harvester (PZH) in this section. The conceptual design to realize the coupled system is depicted in Fig. 1a. The design is similar to that proposed in Maciel et al.^38^ where a circular cylinder is mounted on a spring-damper and piezoelectric system. A pendulum, attached to the cylinder by a rigid rod, is free to rotate about the cylinder with the help of a ball bearing. The rotational damping of the pendulum can be varied by changing the ball bearing. The NRGP-PZH-VIV system can be represented as a schematic in Fig. 1b. It consists of a circular cylinder of mass $m_{{\textrm{s}}}$ and diameter *D* elastically mounted on a spring of stiffness $k_y$ and damper with damping constant $c_y$. A pendulum of mass *M* is pivoted at the centre of the cylinder with length *L* and a rotational damper of constant $c_\theta$. A piezoelectric harvester is also connected to the base of the cylinder, having a resistance $R_y$, capacitance $C_{p,y}$, and electro-mechanical coupling parameter $\theta _y$. When the cylinder is subjected to the incoming freestream velocity of $U_{\infty }$, the cylinder oscillates due to VIV. It is envisaged that the multibody interaction of the cylinder with the pendulum may result in high amplitude oscillation in the de-synchronized regime (internal resonance region), where energy can be harvested.

### Governing equations and non-dimensionalization

The equations of motion of the cylinder-pendulum system can be written by considering the coupling between their motions. The kinetic and potential energy of the cylinder-pendulum system are calculated in order to obtain the Lagrangian of the system and subsequently, the equations of motion are derived on the basis of the Euler-Lagrange equation^35,39^. The governing equations of the NRGP-PZH-VIV system can be written as

1 $$\begin{aligned}&(m_{{\textrm{s}}}+m_{{\textrm{f}}}+M) \frac{d^2 Y}{dt^2}+c_y\frac{dY}{dt}+k_y Y-\theta _yV_y+ ML\bigg (\bigg \{\frac{d\theta }{dt}\bigg \}^2{{\textrm{cos}}}\ \theta +\frac{d^2\theta }{dt^2} {{\textrm{sin}}}\ \theta \bigg ) \nonumber \\&\quad =\frac{1}{2}\rho U_\infty ^2 DLC_{y,v}, \end{aligned}$$

2 $$\begin{aligned}&ML^2\frac{d^2\theta }{dt^2}+c_{\theta }\frac{d\theta }{dt}+ML\bigg (\frac{d^2 Y}{dt^2}+g\bigg ){{\textrm{sin}}}\ \theta =0, \end{aligned}$$

3 $$\begin{aligned}&\frac{d^2 q_y}{dt^2}+\varepsilon _y \omega _f(q_y^2-1)\frac{dq_y}{dt}+\omega _f^2 q_y=\frac{A_y}{D}\frac{d^2 Y}{dt^2}, \end{aligned}$$

4 $$\begin{aligned}&C_{p,y}\frac{dV_y}{dt}+\frac{V_y}{R_y}+\theta _y\frac{dY}{dt}=0, \end{aligned}$$

where Eqs. (1) and (2) depict the coupled equations of motion for the cylinder and the pendulum, respectively. The displacement of the cylinder is denoted by *Y*(*t*) and the angle of rotation of the pendulum is represented by $\theta (t)$. The added mass of the fluid is $m_{{{\textrm{f}}}}$ and the acceleration due to gravity in the transverse direction is *g*. The fluid forces in the transverse direction on the right-hand side of Eq. (1) are represented by the coefficient $C_{y,v} = f(q_y)$. The wake variable $q_y$ is solved using the wake-oscillator model^37^ (Eq. (3)), where an acceleration coupling scheme is utilized on the right-hand side of the equation. Here, in Eq. (3), $A_y$ and $\varepsilon _y$ are the empirically obtained constants of the wake-oscillator model and $\omega _f$ is the vortex-shedding frequency. The energy harvested from the piezoelectric system is evaluated by a constitutive equation that couples the piezoelectric energy generation with the movement of the cylinder^19^, given in Eq. (4) where the voltage is denoted by $V_y$.

Choosing the time scale as $\tau = \omega _{n,y} t$ where $\omega _{n,y}$ is the natural frequency of the structural system in still water, given as

5 $$\begin{aligned} \omega _{n,y}&= 2\pi f_{n,y} = \sqrt{\frac{k_y}{m_{{{\textrm{s}}}} + m_{{{\textrm{f}}}} + M}}, \end{aligned}$$

and the length scale as $y = Y/D$, the coupled dynamics of NRGP-PZH-VIV system, given by Eqs. (1) - (4) can be written in a non-dimensional form as

6 $$\begin{aligned}&\ddot{y}+2\zeta _y {\dot{y}}+y-v_y+\frac{{\overline{m}}}{l_d(1 + C_a/m^*)}\left( {\dot{\theta }}^2{{\textrm{cos}}}\ \theta +\ddot{\theta } {{\textrm{sin}}}\ \theta \right) =\frac{U_r^2}{2\pi ^3}\frac{1}{(m^*+C_a)}C_{y,v}, \end{aligned}$$

7 $$\begin{aligned}&\ddot{q_y}+\varepsilon _y St U_r(q_y^2-1)\dot{q_y}+(St U_r)^2 q_y=A_y\ddot{y}, \end{aligned}$$

8 $$\begin{aligned}&\ddot{\theta }+2\zeta _{\theta }{\dot{\theta }}+( l_d\ddot{y}+\omega _r^2 ){{\textrm{sin}}}\ \theta =0, \end{aligned}$$

9 $$\begin{aligned}&\dot{v_y}+\sigma _{2,y}v_y+\sigma _{1,y}{\dot{y}}=0, \end{aligned}$$

where $\dot{(\ )} = d(\ )/d\tau$ and $\ddot{(\ )} = d^2(\ )/d\tau ^2$ and the dimensionless parameters are defined below:

$$\begin{aligned} \zeta _y&= \frac{c_y}{2(m_{{\textrm{s}}} + m_{{\textrm{f}}} + M)\omega _{n,y}}, \quad v_y = \frac{V_y\theta _y}{V_0}, \quad {\overline{m}} = \frac{M}{M+m_{{\textrm{s}}}}, \\ C_a&= \frac{m_{{\textrm{f}}}}{m_{{\textrm{d}}}}, \quad m^* = \frac{m_{{\textrm{s}}} + M}{m_{{\textrm{d}}}}, \quad l_d = \frac{D}{L}, \\ U_r&= \frac{U_{\infty }}{f_{n,y}D}, \quad St = \frac{\omega _f D}{2\pi U_{\infty }}, \quad \zeta _{\theta } = \frac{c_{\theta }}{2ML^2 \omega _{n,y}}, \\ \omega _r&= \frac{\omega _{n,p}}{\omega _{n,y}}, \quad \sigma _{1,y} = \frac{\theta _y^2}{C_{p,y}(m_{{\textrm{s}}} + m_{{\textrm{f}}} + M)\omega _{n,y}^2}, \quad \sigma _{2,y} = \frac{1}{C_{p,y}R_y\omega _{n,y}}. \end{aligned}$$

Here, the displaced mass of the fluid is denoted by $m_{{\textrm{d}}} = (\pi \rho D^2 {\widetilde{L}})/4$, ${\widetilde{L}}$ being the span of the cylinder. The reference electric tension is represented by $V_0 = (m_{{\textrm{s}}} + m_{{\textrm{f}}} + M)\omega _{n,y}^2 D/\theta _y$. The natural frequency of the pendulum is denoted by $\omega _{n,p}$. Among the non-dimensional parameters, $m^*$ represents the ratio of the combined mass of the cylinder and pendulum to that of the displaced fluid, whereas ${\overline{m}}$ denotes the ratio of the pendulum mass to the combined mass of the cylinder-pendulum system. Note that $\omega _r$, ${\overline{m}}$, $\zeta _\theta$ and $l_d$ describe the coupling between the cylinder and the pendulum, which are crucial for studying the effect of internal resonance on the response of the coupled fluid multibody cylinder-pendulum system.

When the cylinder is considered to be stationary, the right-hand side of Eq. (7) is zero, and thus, solving the equation leads to a limit-cycle periodic solution of wake variable amplitude ${\widehat{q}}_y=2$. The force coefficient in the cross-flow direction ($C_{y,v}$) due to vortex shedding in Eq. (6) can be computed by resolving the fluid force as

10 $$\begin{aligned} C_{y,v}&=\left( C_{L,v}-\frac{C_{D,v}2\pi {\dot{y}}}{U_r}\right) \sqrt{1+\left( \frac{2\pi {\dot{y}}}{U_r}\right) ^2}, \end{aligned}$$

where $C_{L,v}= (q_y/{\widehat{q}}_y){\widehat{C}}_L^o$ is the oscillatory lift coefficient and ${\widehat{C}}_L^o = 0.3842$ is the lift coefficient obtained from the flow around a stationary cylinder^36^. The details of this derivation using the geometric relations can be found in Franzini et al^20^. The drag coefficient is represented by $C_{D,v} = 1.1856$.

Empirical parameters related to wake-oscillator model $(\varepsilon _y\ \text {and}\ A_y)$ are considered from the work of Ogink and Metrikine^36^, wherein two sets of parameters were proposed, viz., for upper-branch ($U_r < 6.5$) and lower-branch ($U_r \ge 6.5$) as follows:

11 $$\begin{aligned} \varepsilon _y = {\left\{ \begin{array}{ll} 0.05,&{}\text {for } U_r< 6.5 \\ 0.7,&{}\text {for } U_r \ge 6.5 \end{array}\right. },\qquad A_y = {\left\{ \begin{array}{ll} 4,&{}\text {for } U_r < 6.5 \\ 12,&{} \text {for } U_r \ge 6.5 \end{array}\right. }. \end{aligned}$$

The upper and lower branches are associated with the higher and lower oscillation amplitudes, respectively. The upper branch represents the synchronized region where the natural frequency of the oscillating system matches with the vortex shedding frequency leading to resonance conditions and thus, higher response amplitudes. Typically, the reduced velocity range of $U_r \in [5, 10]$ is observed in the synchronized region. On the other hand, the lower branch depicts the de-synchronized region where the natural frequency of the system is no more equal to the vortex shedding frequency resulting in absence of resonance and lower amplitudes.

The electric power generation due to the piezoelectric harvester is quantified by the electrical power $P_{el,y}$ and the harvesting efficiency $\eta _{el,y}$ given by^20^

12 $$\begin{aligned} P_{el,y}&= \frac{V_y^2}{R_y}, \end{aligned}$$

13 $$\begin{aligned} \eta _{el,y}&=\frac{P_{el,y}}{(1/2)\rho U_\infty ^3D{\widetilde{L}}} =\frac{4\pi ^4}{U_r^3}\frac{\sigma _{2,y}}{\sigma _{1,y}}(m^*+C_a)v_y^2, \end{aligned}$$

where the expression for efficiency is obtained by making the electric power dimensionless with respect to the flux of fluid kinetic energy across the cylinder frontal area.

### Problem description

In this work, the influence of the NRGP system on the VIV of a cylinder is studied, with a focus on electrical power extraction using piezoelectric materials. In particular, special attention is paid to the interaction between the coupled fluid-multibody-electrical system. The equations for this system (Eqs. (6)-(9)) are solved using fifth-order Runge-Kutta integration based on ordinary differential equation solver in MATLAB, with a fixed time step size of $\Delta t = 0.02$. The non-trivial initial conditions used in these simulations are $q_y(0) = 0.01$ and $\theta (0)=\pi /3$. The simulations are performed till a large non-dimensional time $\tau$ so that the initial transient effects are negligible. In order to understand the coupled multibody dynamics of the cylinder-NRG pendulum system subjected to VIV, four different models are considered, which are as follows:

- *Pure-VIV*: In the literature, Pure-VIV usually refers to a configuration wherein the spring-mounted cylinder system is allowed to undergo VIV freely, without attaching any harvesters. Moreover, the NRG pendulum is also ignored in this case, and therefore, to simulate the Pure-VIV condition, $\sigma _{1,y}=\sigma _{2,y}=v_y={\overline{m}}=0$ in Eqs. (6)-(9).
- *VIV with Piezoelectric harvesters (PZH-VIV)*: In this case, piezoelectric harvesters are considered, however, the effect of NRG pendulum is not included. This is achieved by setting ${\overline{m}}=0$ and $\theta = 0$ in the Eqs. (6)-(9), making it similar to the formulation given in Franzini et al^20^.
- *VIV with NRG pendulum (NRGP-VIV)*: Coupled multibody dynamics of the cylinder-pendulum system subjected to VIV, without including the piezoelectric effects are considered here. Therefore, $\sigma _{1,y}=\sigma _{2,y}=v_y=0$ is substituted in Eqs. (6)-(9) and thereby making it similar to the expressions obtained in Das and Wahi^35^.
- *VIV with NRG pendulum and Piezoelectric harvesters (NRGP-PZH-VIV)*: In this case, the coupled fluid-multibody-electric system is solved to compute the efficiency of the NRG pendulum which is parametrically excited due to VIV. However, since it is a coupled fluid-multibody system, NRGP also influences the cylinder motion, thereby causing fluctuation in fluid forces, which makes it an autoparametric system. Moreover, pendulum parameters are chosen in such a way that the frequency of NRGP is harmonic to the vortex shedding frequency and internal resonance occurs. The effect of internal resonance phenomena on the overall system response and the generated electrical power is the primary focus of this study. Therefore, Eqs. (6)-(9) are solved with the initial conditions stated above.

The parameters for the present study are listed in Table 1. The mass ratio of cylinder-pendulum-fluid system ($m^* = 2.6$) and the structural damping ratio ($\zeta _y = 0.0007$) are selected from the available literature^40^.The added mass coefficient is taken as $C_a = 1$. The effects of the NRG pendulum are incorporated by considering the mass ratio of the cylinder-pendulum system ${\overline{m}} = 0.3$, the ratio of cylinder diameter to the pendulum length $l_d = 0.1$, frequency ratio $\omega _r = 1.3$ and the torsional damping ratio of $\zeta _\theta = 0.0011$. The material parameters for the piezoelectric harvesters are chosen from the work by Mehmood et al.^19^ Here, the numerical simulations are carried out for all the four models mentioned above with a slightly wider range of *Re*, ranging from 1.4 $\times$ 10$^3$ to 2.75 $\times$ 10$^4$.

**Table 1:** Non-dimensional parameters for the NRGP-PZH-VIV system.

Parameter description	Symbol	Value
Linear damping ratio	$\zeta _y$	0.0007
Mass ratio of cylinder-pendulum system	${\overline{m}}$	0.3
Added mass coefficient	$C_a$	1
Mass ratio of cylinder-pendulum-fluid system	$m^*$	2.6
Ratio of cylinder diameter to pendulum length	$l_d$	0.1
Strouhal number	*St*	0.1932
Rotational/torsional damping ratio	$\zeta _{\theta }$	0.0011
Frequency ratio	$\omega _r$	1.3
Dimensionless piezoelectric parameter 1	$\sigma _{1,y}$	0.35
Dimensionless piezoelectric parameter 2	$\sigma _{2,y}$	21.4
Initial angle of pendulum	$\theta _0$	$\pi /3$

A sensitivity analysis is carried out with the aim to investigate the influence of the NRG pendulum parameters on the response of the hydro-elastic multibody system. In particular, the NRG pendulum parameters are chosen in such a way that it triggers an internal resonance. Therefore, the study focuses on the effect of internal resonance on overall response and its possible exploitation to extract more electrical power over a wide range of reduced velocities apart from the lock-in range. It is worth mentioning that the use of a wake-oscillator model to predict the hydrodynamic loads allows for a wider investigation in the sensitivity analysis presented here.

## Comparative study

In this section, a study on the relative performance of all four different models is presented. The results for the Pure-VIV case are compared with the experimental results of Franzini et al.^32,40^ where the influence of pendulum and piezoelectric harvesters are absent. The variation in the oscillation amplitude of the cylinder, hydrodynamic forces, and response frequency are observed and compared for the various models to understand the effect of the introduction of the NRGP on the response of the system. Furthermore, the energy harvesting capability is also compared for the scenarios when the piezoelectric harvester is incorporated, viz., PZH-VIV and NRGP-PZH-VIV.

**Figure 2:**
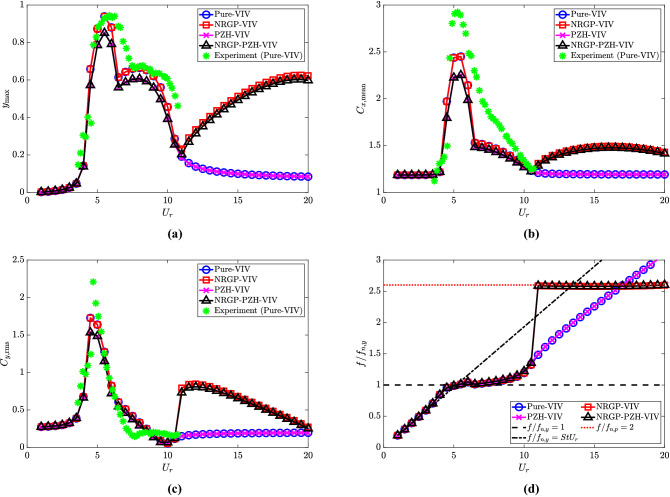
Response characteristics of the system with $U_r$: (**a**) maximum non-dimensional oscillation amplitude ($y_{{\textrm{max}}}$) of the cylinder, (**b**) mean in-line force coefficient $C_{x,{{\textrm{mean}}}}$, (**c**) root-mean-square cross-flow force coefficient $C_{y,{{\textrm{rms}}}}$, and (**d**) dominant frequency with respect to cylinder natural frequency $f/f_{n,y}$. For the NRGP cases, ${\overline{m}} = 0.3$, $l_d = 0.1$, $\omega _r = 1.3$ and $\zeta _\theta = 0.0011$.

The maximum non-dimensional oscillation amplitude ($y_{{\textrm{max}}}$) of the cylinder for all four different models are presented as a function of reduced velocity ($U_r$) in Fig. 2a. In these calculations, the values of non-dimensional parameters $\omega _r = 1.3$, ${\overline{m}} = 0.3$, $l_d = 0.1$ and $\zeta _\theta = 0.0011$ are considered. The experimental results of the Pure-VIV case from the work of Franzini et al.^40^ are also included in the plot for comparison. It can be observed that the response profile of Pure-VIV and PZH-VIV are identical for $U_r \in [0 - 4, 11 - 20]$. The PZH-VIV response for $U_r \in [4 - 11]$ is slightly lesser compared to the Pure-VIV case. Simulation results from Pure-VIV and PZH-VIV cases are in good agreement with the numerical results presented in the work by Franzini et al.^40^ for asymptotic values of NRG pendulum parameters. It should be noted that simulation based on the wake-oscillator model always agree qualitatively with experiments and only captures some features of the phenomenon as fluid forces are computed based on a semi-empirical approach. It uses a set of postulated relations between the empirical parameters, mass ratio, damping, etc. Therefore, the prediction of fluid load using this model has its own limitations which may be the reason for this difference. Therefore, it warrants further investigations on the abidance of empirically assigned wake-oscillator parameters with more experimental data. Another possible extension to the present study can be considered as the modelling of the surrounding fluid domain with the Navier-Stokes equation which will remove the empirically assigned values in the wake-oscillator model, however, these are beyond the scope of this work.

For all four models, a rise in oscillation amplitudes can be observed at $U_r = 4$ and it reaches a peak value at $U_r = 5.5$. This can be attributed to the frequency lock-in, i.e., the synchronization of the vortex shedding frequency with the structural frequency of the system. However, when a NRG pendulum is added to the system, an increase in the peak oscillation amplitude of the cylinder ($y_{{\textrm{max}}}$) is observed at $U_r \ge 11$. This increase of $y_{{\textrm{max}}}$ in the de-synchronized region is due to the internal resonance between the NRG pendulum and the elastically mounted cylinder system. A slight reduction in the oscillation amplitude for the system with piezoelectric harvesters is observed in the response profiles of PZH-VIV (compared to Pure-VIV) and NRGP-PZH-VIV (compared to NRGP-VIV) systems. This reduction can be attributed to the conversion of mechanical to electrical energy.

The in-line and cross-flow force coefficients ($C_x$ and $C_y$, respectively) can also be obtained after solving Eqs. (6)-(8), which are given as

14 $$\begin{aligned} C_x&= \sqrt{1 + \bigg ( \frac{2\pi {\dot{y}}}{U_r} \bigg )^2} \bigg ( C_{D,v} + \frac{2\pi }{U_r}\frac{{\widehat{C}}_L^o}{{\widehat{q}}_y}{\dot{y}} \bigg ), \end{aligned}$$

15 $$\begin{aligned} C_y&= -\frac{2\pi ^3}{U_r^2}C_a \ddot{y} + \sqrt{1 + \bigg ( \frac{2\pi {\dot{y}}}{U_r} \bigg )^2} \bigg ( \frac{{\widehat{C}}_L^o}{{\widehat{q}}_y}q_y - C_{D,v}\frac{2\pi }{U_r}{\dot{y}} \bigg ). \end{aligned}$$

The detailed derivations to obtain in-line and cross-flow force coefficients (by resolving components of drag and lift forces in the transverse and the longitudinal directions) are provided in Ueno and Franzini^33^.

The mean in-line force coefficient ($C_{x, {{\textrm{mean}}}}$) and root-mean-square (rms) cross-flow force coefficient ($C_{y, {{\textrm{rms}}}}$), for the considered models, are also shown in Fig. 2b and c respectively, along with the available measurements of Pure-VIV in Franzini et al.^40^ The variation of $C_{x, {{\textrm{mean}}}}$ for NRGP-VIV is same as Pure-VIV until $U_r = 11$ (Fig. 2b). A jump is observed in the de-synchronized region which is maintained until $U_r = 20$. Similarly for $C_{y, {{\textrm{rms}}}}$ (Fig. 2c), the variation is similar for Pure-VIV and NRGP-VIV cases, till $U_r = 11$, after which a profound jump is observed and reaches a maximum at $U_r = 12$. $C_{y, {{\textrm{rms}}}}$ decreases with further increase in $U_r$. This increase in the force coefficients in the de-synchronized region at $U_r \ge 11$ is associated with the occurrence of internal resonance. The peak values of the present analytical models are lower by about 16$\%$ and 22$\%$ compared to experimental values of $C_{x, {{\textrm{mean}}}}$ and $C_{y, {{\textrm{rms}}}}$, as shown in Fig. 2b and c, respectively. Except for the peak values, the trend of the models matches the experimental measurements satisfactorily.

Figure 2d presents the variation of the non-dimensional response frequency $f/f_{n,y}$ as a function of $U_r$. The frequency lock-in when the vortex shedding frequency equals the structural frequency, $f/f_{n,y} = 1$ is also shown in the figure. The lock-in is observed at $U_r = 4-10$. For Pure-VIV and PZH-VIV models, the dominant frequency follows the Strouhal law beyond the synchronized region. In the case of NRGP models, with further increase in $U_r$ there is a jump in the $f/f_{n,y}$ values observed (see Fig. 2d) from 1.1 to 2.6 at $U_r \ge 11$. This jump is associated with the internal resonance due to the NRG pendulum, which increases the oscillation amplitude in the de-synchronized region, as shown in Fig. 2a. Here, the resonance occurs when the vortex shedding frequency is two times the natural frequency of the pendulum ($f = 2f_{p,y}$), i.e., 2:1 internal resonance, which can be observed in the plot. This 2:1 internal resonance phenomenon may be attributed to the quadratic and cubic nonlinearities due to the transcendental functions introduced into the system by the inclusion of NRGP. A further investigation through methods of multiple-scales and harmonic balance can give more insights into the internal resonance phenomena, however, they are out of the scope of the present work. This 2:1 internal resonance or the frequency lock-in with $f_{n,p}$, starts from $U_r \ge 11$ and continues till $U_r = 20$ for the given set of non-dimensional parameters.

**Figure 3:**
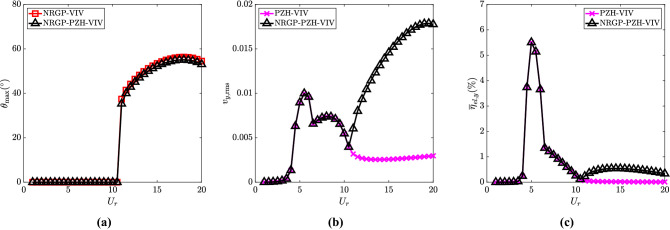
Effect of the introduction of NRGP with $U_r$ on: (**a**) maximum angular rotation of the pendulum $\theta _{{\textrm{max}}}$ in degrees $(^{\circ })$, (**b**) electric tension $v_{y,{{\textrm{rms}}}}$, and (**c**) energy harvesting efficiency ${\overline{\eta }}_{el,y}$. For the NRGP cases, ${\overline{m}} = 0.3$, $l_d = 0.1$, $\omega _r = 1.3$ and $\zeta _\theta = 0.0011$.

To understand the effect of NRGP on VIV, the oscillation of the pendulum in terms of angular position is presented in Fig. 3a as a function of $U_r$. It can be observed that the pendulum oscillation is zero at the synchronized region and starts to oscillate only in the de-synchronized region ($U_r \ge 11$). The oscillation of the pendulum is associated with the internal resonance which occurs in the de-synchronized region. The electrical parameters such as non-dimensional electric tension and piezoelectric harvesting efficiency are associated with the oscillation amplitude of the cylinder and vary with $U_r$. Figure 3b and c present the piezoelectric harvesting capacity for electrical parameters such as rms of electric tension ($v_{y,{{\textrm{rms}}}}$) and efficiency (${\overline{\eta }}_{el,y}$) for $U_r$ ranging from 1 to 20. The pattern of $v_{y,{{\textrm{rms}}}}$ and ${\overline{\eta }}_{el,y}$ are similar for systems with and without NRGP till $U_r = 11$. The difference can be observed for $U_r > 11$. The ${\overline{\eta }}_{el,y}$ is 5.5$\%$ at $U_r = 5$, which is maximum. At the $U_r = 11$ to 20, the efficiency is around 0.6$\%$ for the NRGP system as shown in Fig. 3c.

**Figure 4:**
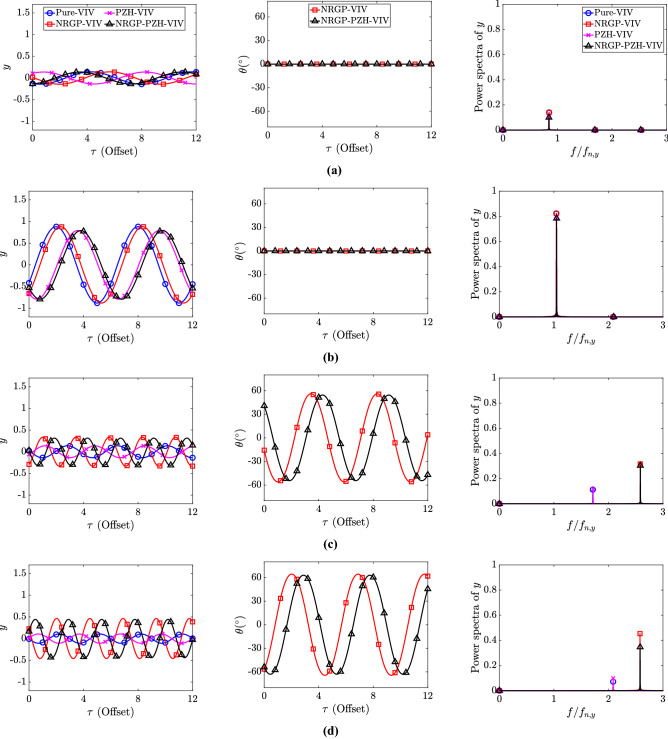
Response amplitude of the cylinder *y* (left) and angular rotation of the pendulum $\theta (^{\circ })$ (middle) and the power spectrum of the amplitude response *y* (right) for various reduced velocity $U_r$: (**a**) 4, (**b**) 6, (**c**) 12, and (**d**) 14. Note that the X-axis in the time history plots has been offset for clarity.

The time histories of the cylinder cross-flow displacement (*y*), angular oscillation of the pendulum, and the frequency spectrum of the cylinder response *y* for all the four models at $U_r \in [4, 6, 12, 14]$ are shown in Fig. 4(Left), (Middle), and (Right), respectively. At $U_r = 4$, the amplitude of the cylinder for Pure-VIV and NRGP-VIV are similar. A slight reduction in amplitude for the system with PZH is observed, as shown in Fig. 4(a-Left). The corresponding pendulum oscillation is zero and the frequency of the cylinder oscillation is close to unity, as shown in Fig. 4(a-Middle and Right). At the synchronization region, i.e., $U_r = 6$, a drastic increase in the amplitude of cylinder response is observed for all cases, however, the response of Pure-VIV and NRGP-VIV is slightly larger compared to PZH systems, as shown in Fig. 4(b-Left). Similar to $U_r = 4$, the pendulum oscillation at $U_r = 6$ is zero and the peak frequency is at the lock-in frequency as expected, i.e., $f/f_{n,y} = 1$, as shown in Fig. 4(b-Middle, Right). With further increase in $U_r$, the system enters the de-synchronized region, where the cylinder oscillation amplitude reduces and an internal resonance is triggered by the pendulum oscillation. As shown in Fig. 4(c-Left), the cylinder oscillations are reduced compared to $U_r = 6$. However, the difference in the introduction of NRGP can be observed clearly as the oscillation amplitude for NRGP-VIV and NRGP-PZH-VIV models are higher compared to non-pendulum models. The pendulum oscillation increases as shown in Fig. 4(c-Middle). The dominant frequency shifts to the higher frequency region, where the vortex shedding frequency is 2 times the pendulum natural frequency, i.e., $f/f_{n,p} = 2$ for the NRGP cases, as shown in Fig. 4(c-Right). Therefore, from the spectrum plots, it is evident that the internal resonance helps in achieving higher oscillation amplitude at higher $U_r$, making the synchronization width more wider compared to the system without NRGP. Pure-VIV and PZH-VIV models follow the Strouhal law in the de-synchronized region. A similar observation can be made for $U_r = 14$ in Fig. 4d where an increase in the cylinder oscillation amplitudes and pendulum oscillations are observed. Therefore, the introduction of NRGP increases the range of $U_r$ where energy extraction can be possible.

## Parametric study of the NRGP-PZH-VIV system

In the previous section, we investigated the effect of the introduction of the pendulum on the VIV response and its energy extraction. Here, we carry out a parametric study to understand the effect of the coupled parameters of the cylinder-pendulum system on the response and piezoelectric harvesting capabilities. A range of frequency-ratio ($\omega _r$), pendulum mass-ratio (${\overline{m}}$), torsional damping-ratio ($\zeta _\theta$), and the ratio of cylinder diameter to pendulum length ($l_d$) are considered to understand their effect on the NRGP-PZH-VIV system. Moreover, the generated electric tension and efficiency are computed and compared with the model without the NRG pendulum (PZH-VIV model).

### Effect of frequency ratio ($\omega _r$)

The frequency ratio $\omega _r$ depicts the ratio of the natural frequencies of the pendulum and the cylinder and is one of the crucial parameters to study the effect of the NRGP on the response of the cylinder in the de-synchronized region. Here, we consider a range of $\omega _r \in [0.5, 1, 1.3, 1.5]$, while all other parameters remain fixed, ${\overline{m}} = 0.3$, $l_d = 0.1$ and $\zeta _\theta = 0.0011$.

**Figure 5:**
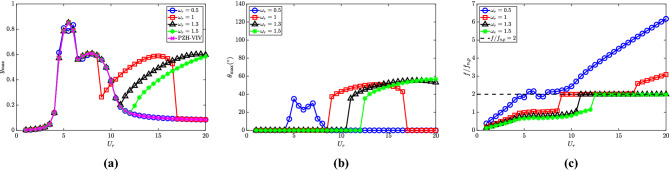
Response characteristics of the system for various $\omega _r$ with $U_r$: (**a**) maximum non-dimensional oscillation amplitude ($y_{{\textrm{max}}}$) of the cylinder, (**b**) maximum angular rotation of the pendulum $\theta _{{\textrm{max}}}$ in degrees $(^{\circ })$ and (**c**) dominant frequency with respect to pendulum natural frequency $f/f_{n,p}$. Here, ${\overline{m}} = 0.3$, $l_d = 0.1$ and $\zeta _\theta = 0.0011$.

The cylinder and pendulum response characteristics for various $\omega _r$ are shown in Fig. 5. The result for the PZH-VIV model is also shown for comparison. When $\omega _r = 0.5$, the oscillation amplitude of the cylinder follows the pattern of PZH-VIV for all $U_r$, as shown in the Fig. 5a. A small dip in the cylinder oscillation amplitude is observed at $U_r = 5$, which can be correlated to the maximum pendulum oscillation observed at the same $U_r$ for $\omega _r = 0.5$ as shown in Fig. 5b. The effect of internal resonance in the de-synchronized region is observed when $\omega _r \ge 1$. The jump in the oscillation amplitude occurs at $U_r = 9$, 11 and 12.5 for $\omega _r = 1$, 1.3 and 1.5, respectively. A quick drop in the amplitude is observed for $\omega _r = 1$ at $U_r = 17$ and the responses are similar to PZH-VIV thereafter, as shown in the figure. For $\omega _r = 1.3$ and 1.5, the oscillation amplitude is higher in the de-synchronized region. In fact, the amplitude values are higher for $\omega _r = 1.3$ compared to 1.5. The pendulum oscillations in the de-synchronized region begin at $U_r = 9$, 11 and 12.5, for $\omega _r = 1$, 1.3 and 1.5 similar to the cylinder oscillation jumps (Fig. 5b). Therefore, the onset of autoparametric excitation of the cylinder response gets delayed with the increase in $\omega _r$ which is reflected by the cylinder as well as the pendulum oscillation amplitudes in Fig. 5a and b, respectively. In Fig. 5a, the response amplitude suddenly drops at $U_r = 17$ for $\omega _r = 1$. It appears that there may be a region of coexisting solutions in the response and this warrants a detailed investigation of the hysteresis phenomenon and/or identifying the basin of attraction (the set of all initial conditions for which internal resonance can be initiated), however, it is beyond the scope of this work.

**Figure 6:**
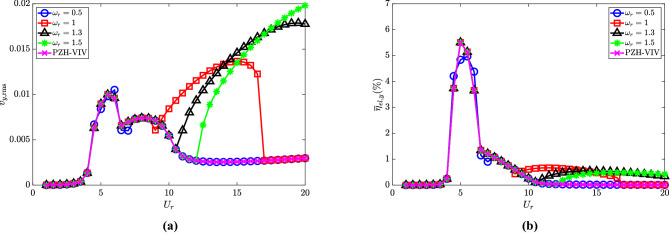
Effect of $\omega _r$ in the introduction of NRGP with $U_r$ on: (**a**) electric tension $v_{y,{{\textrm{rms}}}}$, and (**b**) energy harvesting efficiency ${\overline{\eta }}_{el,y}$. Here, ${\overline{m}} = 0.3$, $l_d = 0.1$ and $\zeta _\theta = 0.0011$.

With the change in $\omega _r$, the natural frequency of the pendulum changes. Therefore, the dominant frequency of oscillation of the cylinder *f* (which is equal to the vortex shedding frequency) is non-dimensionalized by the pendulum frequency $f_{n,p}$ in this scenario, to understand the parametric synchronization due to NRGP in Fig. 5c. It can be observed that the non-dimensionalization collapses the response frequency for various $\omega _r$ along $f/f_{n,p} = 2$. As expected, for $\omega _r = 0.5$, the $f/f_{n,p} = 2$ is equivalent to the VIV synchronization region from $U_r = 4.5 - 8$, and the parametric synchronization does not happen for $U_r \ge 8.5$. For the other cases of $\omega _r = 1$, 1.3 and 1.5, the parametric synchronization occurs at $U_r = 9$, 11 and 12.5, respectively. This corroborated the observations of the response amplitudes of the cylinder and the pendulum. In the case of $\omega _r = 1$, the drop in the cylinder and pendulum oscillation amplitudes can be associated with the deviation of $f/f_{n,p}$ at $U_r = 17$ to 20.

The piezoelectric characteristics for $\omega _r \in [0.5, 1, 1.3, 1.5]$ and PZH-VIV are presented in Fig. 6. The variation of the root-mean-square of the dimensionless electric tension with the reduced velocity is depicted in Fig. 6a. In the case of PZH-VIV model, the peak $v_{y,{{\textrm{rms}}}}$ is 0.01 at $U_r = 5.5$. On attaching the pendulum the electric tension increases in the de-synchronized region, which is more prominent with higher values of $\omega _r$. The effect of the NRGP excitation and the delay in it across $U_r$ with increasing $\omega _r$ is also translated in the variation of $v_{y,{{\textrm{rms}}}}$. The variation of piezoelectric efficiency with $U_r$ is shown in Fig. 6b. It has a maximum efficiency of 5.5$\%$ in all the cases considered at $U_r = 5$. The addition of a pendulum helps in increasing the efficiency to 0.5$\%$ for the NRGP system in the higher $U_r$ region. It should be noted that the electric tension and efficiency for $\omega _r = 0.5$ is similar to PZH-VIV, as the internal resonance occurs at the VIV region for this condition. Therefore, the efficiency is zero in the de-synchronized region.

### Effect of mass ratio (${\overline{m}}$)

The mass ratio (${\overline{m}}$) defined as the ratio of the mass of the pendulum to that of the combined cylinder and pendulum system, is another important parameter in the investigation of the response characteristics of the NRGP system. The effect of ${\overline{m}}$ is investigated by keeping the values of $\omega _r = 1.3$, $l_d = 0.1$ and $\zeta _\theta = 0.0011$ fixed and varying ${\overline{m}} \in [0.1, 0.2, 0.3, 0.5]$.

**Figure 7:**
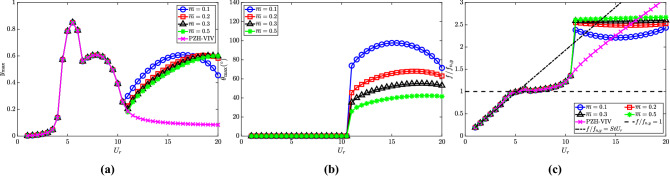
Response characteristics of the system for various ${\overline{m}}$ with $U_r$: (**a**) maximum non-dimensional oscillation amplitude ($y_{{\textrm{max}}}$) of the cylinder, (**b**) maximum angular rotation of the pendulum $\theta _{{\textrm{max}}}$ in degrees $(^{\circ })$ and (**c**) dominant frequency with respect to cylinder natural frequency $f/f_{n,y}$. Here, $\omega _r = 1.3$, $l_d = 0.1$ and $\zeta _\theta = 0.0011$.

The response of the cylinder and pendulum oscillations with the varying mass ratio is shown in Fig. 7. It can be observed that the change in $y_{{\textrm{max}}}$ is small with ${\overline{m}}$, with the maximum displacement occurring for ${\overline{m}} = 0.1$ in the de-synchronized region. Figure 7b presents the variation of pendulum displacement with mass ratio. The initiation of the increase in response amplitude for both the cylinder and the pendulum remains at a similar $U_r$ value for various mass ratios. In the de-synchronized region, at a particular $U_r$, the maximum pendulum oscillation amplitude decreases with an increase in ${\overline{m}}$. The dominant frequency response of the cylinder is shown in Fig. 7c. The VIV lock-in region is identical for all the mass ratios. The parametric excitation as a result of the attached NRGP is observed from $U_r \ge 11$. As $\omega _r = 1.3$ here, the dominant frequency at the de-synchronized region is $2.6f_{n,y}$ which translates to $f/f_{n,p} = 2$. A peculiar behavior is observed for ${\overline{m}} = 0.1$ where the dominant frequency deviates from $f/f_{n,p} = 2$ slightly. Compared to the NRGP model, the PZH-VIV model shows no excitation in the de-synchronized region due to the absence of the pendulum.

**Figure 8:**
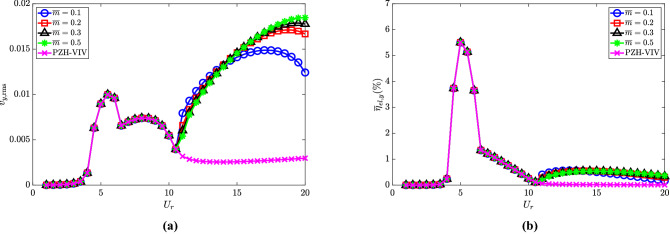
Effect of ${\overline{m}}$ in the introduction of NRGP with $U_r$ on: (**a**) electric tension $v_{y,{{\textrm{rms}}}}$, and (**b**) energy harvesting efficiency ${\overline{\eta }}_{el,y}$. Here, $\omega _r = 1.3$, $l_d = 0.1$ and $\zeta _\theta = 0.0011$.

Figure 8a shows the variation of electric tension with reduced velocity. In the case of PZH-VIV, the peak of 0.01 is observed at $U_r = 5.5$. The introduction of the pendulum in the system increases the maximum rms electric tension $v_{y,{{\textrm{rms}}}}$ by about 42$\%$. With the increase in ${\overline{m}}$, the electric tension also increases. The maximum electric tension estimated is 0.0175 for ${\overline{m}} = 0.5$. The increase in electric tension is attributed to internal resonance in the de-synchronized region. For ${\overline{m}} = 0.1$, the maximum electric tension is observed at $U_r = 17$ and decreases with an increase in $U_r$. The variation of efficiency with $U_r$ is presented in Fig. 8b. It has a maximum efficiency of 5.5$\%$ for systems with NRGP and PZH-VIV cases. The addition of a pendulum helps to achieve higher efficiency of 0.5$\%$ at $U_r \ge 11$, as shown in the figure. However, the influence of the mass ratio on the efficiency is observed to be negligible.

### Effect of torsional damping ratio ($\zeta _{\theta }$)

The effect of the torsional damping ratio ($\zeta _\theta$) for the NRG pendulum on the autoparametric excitation of the cylinder is studied in this sub-section. Four representative values of the damping ratio are considered, viz., $\zeta _\theta \in [2.75\times 10^{-4}, 1.1\times 10^{-3}, 4.4 \times 10^{-3}, 1.76 \times 10^{-2}]$. The other crucial parameters are held constant at ${\overline{m}} = 0.3$, $l_d = 0.1$ and $\omega _r = 1.3$.

**Figure 9:**
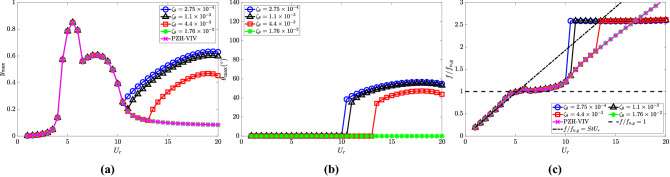
Response characteristics of the system for various $\zeta _\theta$ with $U_r$: (**a**) maximum non-dimensional oscillation amplitude ($y_{{\textrm{max}}}$) of the cylinder, (**b**) maximum angular rotation of the pendulum $\theta _{{\textrm{max}}}$ in degrees $(^{\circ })$ and (**c**) dominant frequency with respect to cylinder natural frequency $f/f_{n,y}$. Here, ${\overline{m}} = 0.3$, $l_d = 0.1$ and $\omega _r = 1.3$.

The response characteristics of the cylinder and the pendulum depicting the effects of the damping ratio are shown in Fig. 9. The torsional damping is observed to affect the initiation of the autoparametric excitation. It occurs at $U_r = 10.5$, 11 and 13 for $\zeta _\theta = 2.75 \times 10^{-4}$, $1.1 \times 10^{-3}$ and $4.4 \times 10^{-3}$, respectively. However, there is no internal resonance observed for the high damping value of $\zeta _\theta = 1.76 \times 10^{-2}$ and the cylinder response follows the PZH-VIV model (Fig. 9a). The maximum oscillation amplitudes for the cylinder and the pendulum also decrease for higher $\zeta _\theta$ values. The delay in the initiation of internal resonance with respect to $U_r$ as $\zeta _\theta$ increases is confirmed by the dominant frequency plot in Fig. 9c. This delay also indicates that the energy extraction window reduces with an increase in $\zeta _\theta$ for the range of $U_r$ considered in the study.

**Figure 10:**
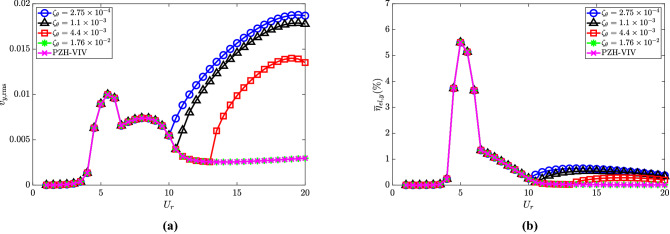
Effect of $\zeta _\theta$ in the introduction of NRGP with $U_r$ on: (**a**) electric tension $v_{y,{{\textrm{rms}}}}$, and (**b**) energy harvesting efficiency ${\overline{\eta }}_{el,y}$. Here, ${\overline{m}} = 0.3$, $l_d = 0.1$ and $\omega _r = 1.3$.

The dimensionless electric tension and the energy harvesting efficiency for varying $\zeta _\theta$ are shown in Fig. 10a and b, respectively. The electric tension is maximum for the lowest torsional damping ratio, i.e., $\zeta _\theta = 2.75\times 10^{-4}$ as observed in the internal resonance region. A reduction in the electrical tension is observed with an increase in torsional damping. Similar to the cylinder oscillation amplitude, the electric tension at $\zeta _\theta = 1.76 \times 10^{-2}$ follows the PZH-VIV trend for all the $U_r$ considered in this study. In Fig. 10b, for lower values of $\zeta _\theta$ there is a rise in efficiency in the internal resonance region. The maximum efficiency achieved for all four cases is 5.8$\%$ at $U_r = 5$ (VIV region) and in the de-synchronized region, the maximum efficiency is about 0.5$\%$. These results are quite intuitive as an increase in the torsional damping will tend to dampen out the oscillations of the pendulum leading to negligible effects on the autoparametric excitation. Therefore, the torsional damping is supposed to be kept at a lower value for obtaining the benefits of NRGP autoparametric excitation in the de-synchronized region.

### Effect of ratio of cylinder diameter to pendulum length ($l_d$)

Finally, the effect of the ratio of cylinder diameter *D* to the length of the pendulum *L* on the internal resonance of the NRGP-PZH-VIV system is investigated in this sub-section. To accomplish this, we consider $l_d \in [0.1, 0.3, 0.5]$ while keeping the other parameters constant at ${\overline{m}} = 0.3$, $\omega _r = 1.3$ and $\zeta _\theta = 0.0011$.

**Figure 11:**
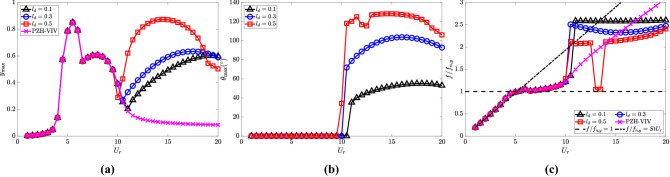
Response characteristics of the system for various $l_d$ with $U_r$: (**a**) maximum non-dimensional oscillation amplitude ($y_{{\textrm{max}}}$) of the cylinder, (**b**) maximum angular rotation of the pendulum $\theta _{{\textrm{max}}}$ in degrees $(^{\circ })$ and (**c**) dominant frequency with respect to cylinder natural frequency $f/f_{n,y}$. Here, ${\overline{m}} = 0.3$, $\omega _r = 1.3$ and $\zeta _\theta = 0.0011$.

The cylinder oscillation amplitude $y_{{\textrm{max}}}$ for $l_d = 0.5$ is observed to be maximum compared to other $l_d$ values as shown in Fig. 11a. The maximum cylinder oscillation achieved in the de-synchronized region is at $U_r = 15$, which is equal to the amplitude estimated at $U_r = 6$. It is worth noting that the highest oscillation amplitude achieved in the de-synchronized region is for the NRGP system with $l_d = 0.5$, ${\overline{m}} = 0.3$, $\omega _r = 1.3$ and $\zeta _\theta = 0.0011$ compared to other parametric investigations discussed earlier. Similar to the cylinder oscillation amplitude, the maximum pendulum oscillation is achieved for $l_d = 0.5$, as shown in Fig. 11b. It can be observed that the range of $U_r$ values for getting the autoparametric excitation increases with an increase in $l_d$. The $f/f_{n,y}$ values for $l_d = 0.1$, 0.3 and 0.5 are presented in Fig. 11c. The $f/f_{n,y}$ increases at $U_r = 11$ till $U_r = 12.5$ for $l_d = 0.5$. Then the $f/f_{n,y}$ drops at $U_r = 13$ and 13.5 close to the VIV lock-in frequency. The $f/f_{n,y}$ again increases to at $U_r = 14$ till $U_r = 20$. The drop in the $f/f_{n,y}$ values at $U_r = 13$ and 13.5 reflects the lock-in behavior as in the case of the synchronized region.

**Figure 12:**
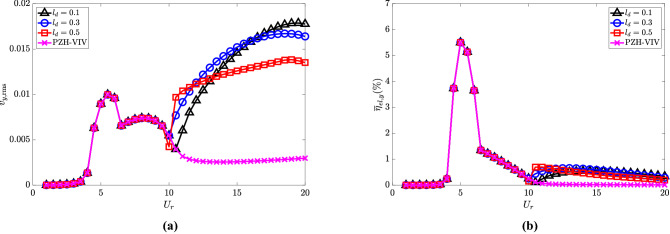
Effect of $l_d$ in the introduction of NRGP with $U_r$ on: (**a**) electric tension $v_{y,{{\textrm{rms}}}}$, and (**b**) energy harvesting efficiency ${\overline{\eta }}_{el,y}$. Here, ${\overline{m}} = 0.3$, $\omega _r = 1.3$ and $\zeta _\theta = 0.0011$.

The electric tension and efficiency are presented in Fig. 12a and b, respectively. The maximum electric tension is observed for $l_d = 0.1$ at $U_r = 20$, and it decreases with an increase in $l_d$ as shown in Fig. 12a at high $U_r$. As shown in Fig. 12b, the maximum efficiency of all three cases is computed to be 5.5$\%$ and 0.5$\%$ in the synchronized and de-synchronized regions, respectively.

## Conclusions

In this work, a VIV-based device for electrical energy extraction using a piezoelectric harvester is proposed in which a non-linear rotative gravity pendulum (NRGP) is attached. It is observed that the addition of a pendulum to a cylinder undergoing cross-flow VIV increases the maximum electrical output nearly by four times more than that of a device without the pendulum. A significant increase in the cylinder displacement can be observed for reduced velocity greater than 10, i.e, in the de-synchronized region. This can be attributed to the 2:1 internal resonance which may be due to the quadratic and cubic nonlinearities introduced into the multibody system through the inclusion of a rotating pendulum. The coupled cylinder-pendulum parameters $\omega _r$, ${\overline{m}}$, $\zeta _\theta$, and $l_d$ play an important role in the energy harvesting capability of the system. Some of the key findings from the current study are:

- The presence of the non-linear rotative gravity pendulum results in the internal resonance of the cylinder-pendulum system at the de-synchronized regime ($U_r > 11$) where the cylinder oscillates with a dominant frequency twice the pendulum’s natural frequency.
- The onset of the autoparametric excitation gets delayed in terms of $U_r$ with an increase in $\omega _r$ and $\zeta _\theta$. It remains at a similar $U_r$ value for various ${\overline{m}}$ and gets advanced with an increase in $l_d$.
- The piezoelectric harvesting efficiency is observed to be higher in the de-synchronized region compared to the case without the pendulum.

A systematic study through perturbation and/or continuation methods to identify the parameter space of internal resonance is of significant importance to accurately predict/control the system dynamics. Moreover, instead of modelling the fluid forces with a reduced order wake-oscillator model, the accuracy can further be improved by modelling the fluid domain with incompressible Navier-Stokes equations along with turbulent fluctuations/noise and attempting to solve a fully coupled fluid-solid multibody system. Physical experiments of the NRGP-PZH-VIV model and the possibility of harvesting energy for the NRGP-VIV system considering electrostatic/electromagnetic extraction and their comparison can be explored in future research.

## Data Availability

The data that support the findings of this study are available from the corresponding author upon reasonable request.
